# Resolutions of Respect to Dr. Theodore Emanuel Le Caudey

**Published:** 1901-08

**Authors:** 


### Obituary.

### RESOLUTIONS OF RESPECT TO DR. THEODORE EMANUEL LE CAUDEY.

Whereas, We, the members of the American Dental Society of Europe, have heard with deep regret of the death of our distinguished colleague, Dr. Theodore Emanuel Le Caudey; therefore

*Resolved,* That by this sad event our profession has lost one of its most revered members, whose fame belonged not only to his own country, but which was also the prized possession of the civilized world.

*Resolved,* That we offer to his afflicted family the assurances of our respectful sympathy.

W. E. Royce.
N. S. Jenkins.
W. R. Patton.

